# Assessment of two different HER2 scoring systems and clinical relevance for colorectal cancer

**DOI:** 10.1007/s00428-019-02668-9

**Published:** 2019-11-13

**Authors:** Furong Liu, Chao Ren, Ying Jin, Shaoyan Xi, Caiyun He, Fang Wang, Zixian Wang, Rui-hua Xu, Feng Wang

**Affiliations:** 1 Sun Yat-sen University Cancer Center, State Key Laboratory of Oncology in South China, Collaborative Innovation Center for Cancer Medicine, 651 Dongfeng Road East, Guangzhou, 510060 People’s Republic of China; 2 Department of Medical Oncology, Sun Yat-sen University Cancer Center, 651 Dongfeng Road East, Guangzhou, 510060 People’s Republic of China; 3 Department of Pathology, Sun Yat-sen University Cancer Center, 651 Dongfeng Road East, Guangzhou, 510060 People’s Republic of China; 4 Department of Molecular Diagnostics, Sun Yat-sen University Cancer Center, 651 Dongfeng Road East, Guangzhou, 510060 People’s Republic of China

**Keywords:** HER2, Colorectal cancer, Methodology, Prognosis

## Abstract

Although the positivity of human epidermal growth factor receptor 2 (HER2) is low in colorectal cancer (CRC), anti-HER2 is becoming a new target therapy in metastatic colorectal cancer (mCRC). However, assessment of the HER2 scoring system was still not established in CRC. The purpose of our study was to evaluate HER2 status and its correlation with clinicopathological characteristics and survival according to the HER2 diagnostic criteria for gastroesophageal adenocarcinoma (GEA criteria) and the HERACLES diagnostic criteria (HERACLES criteria) in a large cohort of Chinese CRC patients. The HER2 positivity was 2.9% (43/1490) and 2.6% (39/1490) in CRCs based on the GEA criteria and the HERACLES criteria, and 3.7% (9/243) in mCRC according to both criteria. HER2 status was associated with primary tumor location (*P* = 0.037), regional lymph node metastasis (*P* = 0.035), and TNM stage (*P* = 0.022) in CRCs based on the HERACLES criteria. No such association was found based on the GEA criteria. Furthermore, HER2 positive only presented in patients with RAS gene wild type (*P* = 0.001). Significant difference was only observed between the HER2-positive and HER2-negative groups in terms of disease-free survival for stage II-III CRCs (*P* = 0.048) according to the HERACLES criteria, but not based on the GEA criteria. Our findings suggest that the frequency of HER2 overexpression or amplification was low in Chinese CRC patients, and provide a rationale for further evaluation of HER2 in CRC based on the HERACLES criteria and the HER2 diagnostic criteria for gastroesophageal adenocarcinoma.

**Electronic supplementary material:**

The online version of this article (10.1007/s00428-019-02668-9) contains supplementary material, which is available to authorized users.

## Introduction

Colorectal cancer (CRC) is the third most common malignant neoplasm and the second leading cause of cancer-related deaths worldwide [1]. It is estimated that 370,000 newly diagnosed cases of CRC and 180,000 deaths from CRC occurred in China in 2014 [2]. Approximately 20% of the patients with newly diagnosed CRC already have distant metastases at the time of diagnosis and for those without metastasis [3], they can be treated with curative approaches but still retain a high risk of recurrence (up to 50%) [4]. As such, advanced stage diseases and recurrences after curative treatment are a significant cause of death. The standard treatment for such patients is chemotherapy, and recently, the addition of targeted therapies has shown improved therapeutic outcomes. Cetuximab and panitumumab, which are directed against the epidermal growth factor receptor (EGFR), are the most common monoclonal antibody used in metastatic colorectal cancer (mCRC) to prolong survival [4, 5]. However, approximately 50% of CRC patients cannot benefit from the anti-EGFR treatments since their tumors harbor the RAS or BRAF mutations [6, 7]. The biology of CRC is still far from being fully understood, and researchers are still striving to identify new biomarkers for potential therapeutic targets and novel approaches for predicting therapeutic responses and improving survival outcomes.

The human epidermal growth factor receptor 2 (HER2, also known as HER2/neu, C-erbB2, and p185) is a member of the EGFR family of receptor tyrosine kinases [8]. Over the past two decades, the status of HER2 has shown to have an important role in the development and progression of approximately 30% of breast cancer cases and 10% of gastric cancer cases [9–11]. Trastuzumab is the monoclonal antibody that acts by blocking the HER2 receptor, and its use has become a standard treatment for patients with breast and gastric cancer presenting with HER2 gene amplification or membranous HER2 protein overexpression [12, 13]. After the breakthroughs in breast and gastric cancers, the efficacy of anti-HER2 therapy was evaluated in CRC. The HERACLES trial was a multicenter open-label phase II trial that enrolled patients with wild-type KRAS exon 2, HER2 overexpressing metastatic colorectal cancer refractive to chemotherapy, and anti-EGFR therapy resistance. The objective response rate (ORR) was 30% for patients received a combination of trastuzumab and lapatinib [14]. Therefore, they brought up criteria for HER2 assessment in CRC (HERACLES criteria). However, until present, other studies also used the HER2 diagnostic criteria for gastroesophageal adenocarcinoma (GEA criteria) for HER2 assessment in CRC [15–17]. Moreover, the relationship between the expression of HER2 and clinicopathological prognostic factors remains controversial [15, 17–20]. These debatable results indicate that the role of HER2 in CRC requires further exploration.

Thence, the purpose of this study was to compare the two different HER2 scoring systems of CRC in large Chinese patients and analyze the influence of HER2 status on clinicopathological factors and survival of CRC.

## Material and methods

### Study design

One thousand five hundred sixty CRC patients with formalin-fixed paraffin-embedded archival samples at Sun Yat-sen University Cancer Center from January 2016 to December 2017 were identified. One thousand five hundred fifteen patients enrolled by fulfilling the following inclusion criteria: (a) patients diagnosed as histologically prove CRC; (b) patients with complete clinical, pathological, and prognostic information. Twenty-five patients were excluded based on the following exclusion criteria: (a) lack of tumor tissue for further test (five cases); (b) presence of simultaneous carcinoma (11 patients); (c) refusal anti-tumor treatment (nine patients). Finally, 1490 eligible patients were enrolled in our analysis (Supplementary Fig. 1). The institutional review board approved the study, and informed consent was waived. All clinicopathologic data were obtained from the patients’ records.

### HER2 expression and amplification tests

HER2 expression analysis was performed by immunohistochemistry (IHC) using primary monoclonal antibody against HER2/neu (clone CB-11, dilution 1:65, Cell Marque, Rocklin, CA, USA), following the manufacturer’s protocol. The procedure was carried out in our Department of Pathology. All tumor tissue specimens were processed by the hematoxylin and eosin (H&E) method, and the sections comprising at least 70% tumor cells were cut into 4-μm sections for IHC staining. The HER2 immunoreactivity was presented with percentage and intensity. The intensity of reactivity was grouped as IHC score 0, no reactivity or membrane staining; IHC score 1+, faint/barely perceptible partial membrane staining; IHC score 2+, weak to moderate complete or basolateral membrane staining; and IHC score 3+, moderate to strong complete membrane staining [21]. IHC analysis was performed by one trained pathologist. Ambiguous cases were reanalyzed by a second pathologist.

HER2 amplification analysis was performed by fluorescent in situ hybridization (FISH) using the FDA-approved Vysis PathVysion HER-2/neu DNA Probe Kit (Dako Cytomation, Denmark). The scoring was carried out in no less than 20 non-overlapping nuclei core in tumor regions. A ratio of HER2 signal to CEP17 signal of ≥ 2 was considered amplification of HER2 [21].

### Assessment of HER2 status

#### The HER2 diagnostic criteria for gastroesophageal adenocarcinoma (GEA criteria)

HER2-positive cases were those who had an IHC score of 3+ in more than 10% of the tumor cells or for those who had an IHC score 2+ in more than 10% of the tumor cells and demonstrated positive amplification of HER2 gene by FISH [21].

#### The HERACLES diagnostic criteria (HERACLES criteria)

The HERACLES criteria of HER2 positive were tumors with a 3+ HER2 score in more than 50% of the tumor cells by IHC or with a 3+ HER2 score in 10~50% of the tumor cells by IHC and FISH positive, or with a 2+ HER2 score two in more than 50% of the tumor cells by IHC and FISH positive. FISH positivity was defined as a HER2:CEP17 ratio higher than two in more than 50% of the tumor cells [14].

### Follow-up

All patients underwent continuous follow-up in accordance with that specified in the National Comprehensive Cancer Network (NCCN) guidelines. The disease-free survival (DFS) for patients with stage II–III was defined as the time from the first day of surgery until the first documented recurrence date, or death from any cause or the time censored. The progression-free survival (PFS) for mCRC patients was defined as the time from the first day of treatment until the first documented progressive disease (PD) according to Response Criteria Evaluation in Solid Tumors (RECIST) version 1.1 or death from any cause, whichever occurred first for mCRC [22]. The censor date was December 31, 2018.

### Statistical analysis

Comparisons were made using *t* test, the chi-square test, and Fisher exact test, as appropriate. The survival was analyzed using the Kaplan-Meier method and the log-rank test was used to determine the significance of the difference between the survival curves. Univariate analysis and multivariate analysis were performed by the Cox proportional hazards regression model to determine the significant prognostic factors on survival. All tests were two-tailed, and statistical significance was set at *P* < 0.05. The statistical analysis of the data was performed using SPSS 19.0 statistical software (SPSS, Chicago, IL, USA).

## Results

### Clinicopathological characteristics of CRC

A total of 1490 cases of colorectal cancer were included. Of all investigated patients, there were 933 (62.6%) males and 557 (37.4%) females. The median age was 60 years (range 16–91 years). Six (0.4%) specimens were from metastatic sites, while 1484 (99.6%) were from primary tumor sites through colectomy or colonoscopic biopsy. Regarding the location of the primary tumor, 1110 (74.5%) tumors on the left side, 365 (24.5%) tumors on the right side, and 15 (1.0%) tumors on both sides of the colon. Histopathological examination identified 1435 (96.3%) and 55 (3.7%) cases with well to moderately, and poorly differentiated tumors, respectively. A total of 650 patients (43.6%) were stage I and II, and 840 patients (56.4%) were stage III and IV according to the 7th American Joint Committee on Cancer (AJCC)/International Union Against Cancer (UICC) staging system (Table 1).

**Table 1:** Relationship between the expression of HER2 and the clinicopathological characteristics of colorectal cancer according to the two criteria

Variables	The criteria for gastroesophageal adenocarcinoma				The HERACLES diagnostic criteria		
	All patients	HER2 negative	HER2 positive	*P* value	HER2 negative	HER2 positive	*P* value
Gender				0.149			0.179
Male	933	911	22		913	20	
Female	557	536	21		538	19	
Age (years)				0.867			0.860
≤ 65	1050	1019	31		1023	27	
> 65	440	428	12		428	12	
Primary tumor location							
Left-sided	1110	1073	37	0.108 ^a^	1075	35	*0.037*^a^
Right-sided	365	359	6		361	4	
Both-sided	15	15	0		15	0	
Differentiation grade				1.0			1.0
Well-moderate	1435	1393	42		1397	38	
Poor	55	54	1		54	1	
Regional lymph node metastasis							
No. of cases evaluated	1429 ^b^			0.062			*0.035*
Absent	681	667	14		669	12	
Present	748	719	29		721	27	
TNM stage				0.086			*0.022*
I–II	650	637	13		640	10	
III–IV	840	810	30		811	29	
MSI test							
No. of cases evaluated	1452 ^c^			0.247			0.111
MSI-H (MMR-D)	126	125	1		126	0	
MSS/MSI-L (MMR-P)	1326	1289	37		1292	34	
RAS gene							
No. of cases evaluated	201^d^			*0.001*			*0.001*
RAS wild type	112	101	11		101	11	
RAS mutation	89	89	0		89	0	
Ki-67							
No. of cases evaluated	1423 ^e^			0.507			0.463
≤ 15%	88	85	3		85	3	
> 15%	1335	1300	35		1304	31	

^a^*P* was evaluated between left and right sided

^b^Regional lymph node metastasis of 58 cases was difficult to identify due to the neoadjuvant chemotherapy or/and radiotherapy (3 cases) and at stage IV disease (58 cases)

^c^38 cases did not receive MSI test

^d^201 cases received RAS gene test

^e^67 cases did not receive Ki-67 test

*HER2*, human epidermal growth factor receptor 2; *MSI*, microsatellite instability; *MSI-H*, high-level microsatellite instability; *MMR-D*, mismatch repair deficiency; *MSS*, microsatellite stability; *MSI-L*, low-level microsatellite instability; *MMR-P*, mismatch repair proficiency

We further analyzed clinicopathological characteristics of mCRC. For 244 patients with mCRC, 150 (61.5%) were male, and 94 (38.5%) were female; the median age of this cohort was 58 years (range 23–85 years). A total of 163 (66.8%) patients presented with liver metastases, making it the most common metastatic site. A total of 146 (59.8%) patients received an RAS gene test, and 58 (39.7%) of them were found to harbor RAS mutations. A total of 228 (93.4%) patients underwent DNA mismatch repair or microsatellite instability tests, and 10 (4.4%) cases displayed DNA mismatch repair deficiency (MMR-D) or high-level microsatellite instability (MSI-H) phenotypes (Supplementary Table 1).

### HER2 assessment in CRC

#### The GEA criteria

**Fig. 1:**
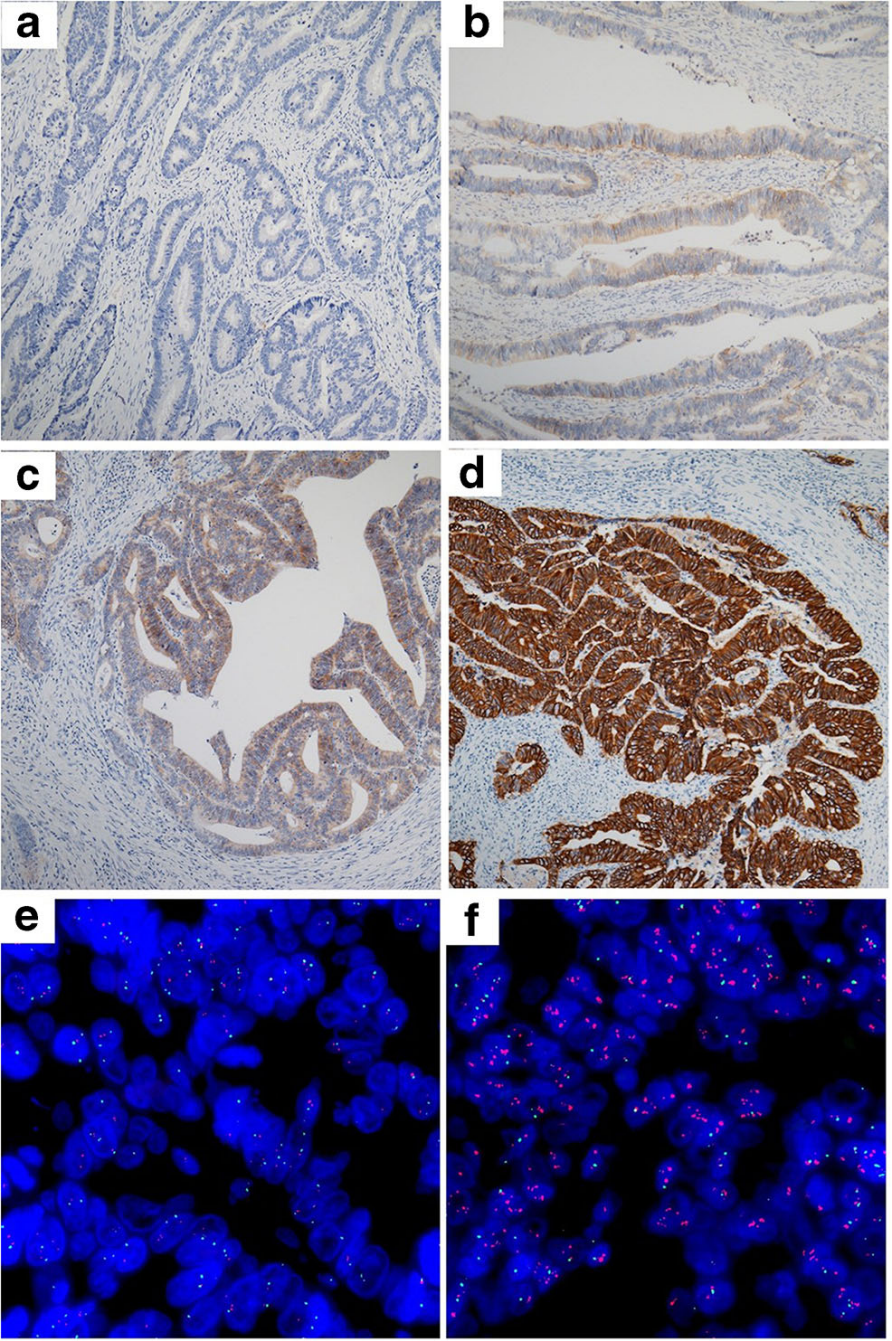
HER2 evaluation by immunohistochemistry and fluorescence in situ hybridization in colorectal cancer specimens. Representative immunostaining intensity of tumor cells: 0/ none (**a**), 1+/ faint (**b**), 2+/ week to moderate (**c**), 3+/ strong (**d**). Representative negative (**e**) and positive (**f**) amplification of HER2 gene

Of the 1490 CRC specimens assessed by IHC, 959 (64.4%), 410 (27.5%), 87 (5.8%), and 34 (2.3%) had HER2 scores of 0, 1+, 2+, and 3+, respectively, according to the criteria for gastroesophageal adenocarcinoma (Fig. 1a–d). FISH was performed for the 87 cases with IHC score 2+, of which 78 cases had negative amplification of the HER2 gene, and 9 cases had positive amplification of the HER2 gene (Fig. 1e, f). Overall, 1447 (97.1%) cases were evaluated as HER2 negative, and 43 (2.9%) cases were HER2 positive (Table 2). For the mCRC cohort, 167 (68.4%), 56 (23.0%), 12 (4.9%), and 9 (3.7%) cases assessed by IHC showed HER2 scores of 0, 1+, 2+, and 3+, respectively. FISH examination for the 12 cases of HER2 score 2+ showed that only one case had positive amplification of the HER2 gene. In total, 234 (95.9%) cases were evaluated as HER2 negative and only 10 (4.1%) cases were HER2 positive (Supplementary Table 2).

**Table 2:** HER2 assessment in colorectal cancer according to different systems

		The criteria for gastroesophageal adenocarcinoma		The HERACLES diagnostic criteria	
HER2 (IHC)	Valid/missing	1490/0		1490/0	
	0	959	64.4%	959	64.4%
	1+	410	27.5%	410	27.5%
	2+	87	5.8%	87	5.8%
	3+	34	2.3%	34	2.3%
HER2 (FISH)	Valid/missing	87/1403		91/1399	
	Not amplified	78	89.7%	82	90.1%
	Amplified	9	10.3%	9	9.9%
HER2 status	Valid/missing	1490/0		1490/0	
	Negative	1447	97.1%	1451	97.4%
	Positive	43	2.9%	39	2.6%

*HER2*, human epidermal growth factor receptor 2; *IHC*, immunohistochemistry; *FISH*, fluorescent in situ hybridization

#### The HERACLES criteria

Further, FISH was performed for 4 cases with a HER2 score 3+ but in less than 50% of the tumor cells, as determined by IHC, according to the colorectal cancer-specific HERACLES diagnostic criteria [14], and the FISH results were all negative. We checked the 4 cases, two of them were with a 3+ HER2 score in 10% of the tumor cells by IHC, the intensity of the third one was moderate but also classified into 3+ score after discussion and the percentage was 30% of the tumor cells, and the fourth one was with a 3+ HER2 score in 40% of the tumor cells by IHC. Owing to the heterogeneity of tumor and the definition of HER2 amplification by FISH according to the HERACLES criteria, the 4 cases were classified to HER2 negative. Among the 4 cases, there was one patient with stage I, two patients with stage II, and one patient with stage III disease. Hence, 2.6% (39/1490) of cases showed HER2 positivity in all CRCs according to the HERACLES criteria (Table 2). The positivity of HER2 for mCRC cohort was 4.1% (10/244), the same according to both criteria (Supplementary Table 2).

### Correlation of HER2 expression with clinicopathological characteristics in all CRCs

#### The GEA criteria

The HER2 positivity rate was more common in female patients (37.7% vs. 23.6%); however, the difference was not statistically significant (*P* = 0.149). In that HER2 positivity only presented in patients with RAS wild type, HER2 status was significantly correlation with RAS gene (*P* = 0.001). However, it was no correlation between HER2 status with clinicopathological variables such as primary tumor location (*P* = 0.108), tumor differentiation grade (*P* = 1.0), and TNM stage (*P* = 0.086) in all CRCs according to the GEA criteria (Table 1).

#### The HERACLES criteria

According to the HERACLES criteria, HER2 positivity was significantly correlation with primary tumor location (*P* = 0.037), regional lymph node metastasis (*P* = 0.035), TNM stage (*P* = 0.022), and RAS status (*P* = 0.001) (Table 1).

### Correlation of HER2 expression with clinicopathological characteristics in mCRCs

Since the positivity was the same according to both criteria, the association of HER2 status and clinicopathological variables was further explored in mCRC. One hundred forty-six patients received an RAS gene test, and HER2 positivity was statistically significant associated with RAS gene (*P* = 0.042). Yet the results were not significant for primary tumor site (*P* = 0.122) and metastatic site (*P* = 0.5) (Supplementary Table 1).

### Survival analysis

**Fig. 2:**
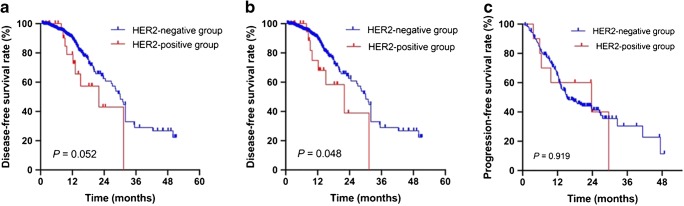
Kaplan-Meier curves of disease-free survival in HER2-positive and HER2-negative patients with stage II-III disease treated with surgery according to the HER2 diagnostic criteria for gastroesophageal adenocarcinoma (**a**) and the HERACLES diagnostic criteria (**b**). Kaplan-Meier curves of progression-free survival in HER2-positive and HER2-negative patients with metastatic colorectal cancer (**c**)

The median follow-up time was 11.1 months (range 2.6–65.4 months). DFS was evaluated in patients with stage II-III CRC who were treated with surgery. No significant difference was observed between the HER2-positive and HER2-negative groups in terms of DFS either according to the GEA criteria (*P* = 0.052) (Fig. 2a). However, HER2 status was associated with DFS according to the HERACLES criteria (*P* = 0.048) (Fig. 2b), yet multivariate analysis showed that HER2 status was not an independent prognosis for DFS (Supplementary Table 3).

RFS was evaluated in patients with mCRC. A total of 127 patients presented with PD after first-line treatment during the follow-up period. The median PFS was 13.3 months for all mCRCs. The median PFS was 15.4 months for patients with HER2 negativity, while it was 23.7 months in patients with HER2 positivity. No significant difference was observed between the HER2-positive and HER2-negative groups in terms of PFS in mCRC (*P* = 0.919) (Fig. 2c).

## Discussion

In this large Chinese cohort study, we evaluated HER2 status in CRC based on two different criteria and found that HER2 positivity in colorectal adenocarcinoma was quite low in Chinese population, with a rate of 2.9% according to the HER2 diagnostic criteria for gastroesophageal adenocarcinoma, and a rate of 2.6% according to the HERACLES criteria. HER2 status was associated with clinical variables and DFS in patients with stage II-III CRC according to the HERACLES criteria, yet, the results were not found according to the HER2 diagnostic criteria for gastroesophageal adenocarcinoma. However, HER2 status had no influence on RFS in patients with mCRC.

Various research groups have investigated the frequency of HER2 expression in CRC, with a variability ranging from 1.3 to 82% owing to different scoring systems [15–17]. Richman SD et al. [15] analyzed HER2 amplification/overexpression in 3256 patients from three colorectal cancer trials in the UK and found that the overexpression of HER2 was observed in 25 of 1914 (1.3%) stage II-III tumors and 29 of 1342 (2.2%) stage IV tumors. This small observed proportion of HER2 expression is in line with our obtained results for CRC. Since the standard of HER2 evaluation criteria was not established in CRC, the scoring system of HER2 in CRC of these studies usually used the criteria in gastroesophageal adenocarcinoma (GEA) [15–17, 23].

Valtorta E et al. [24] developed the HERACLES diagnostic criteria for HER2 in CRC: tumors with a 3+ HER2 score in more than 50% of cells by IHC or tumors with a 2+ HER2 score and a HER2:CEP17 ratio higher than two in more than 50% of cells by FISH. The significant difference from the criteria in GEA lies in the cutoff value for the IHC evaluation. Our study was the first to evaluate the two different scoring systems of HER2 for CRC in one large cohort. Further, we found HER2 positivity was associated with tumor location (*P* = 0.037), regional lymph node metastasis (*P* = 0.035), and tumor stage (*P* = 0.022) according to the HERACLES criteria, which were not present according to the GEA criteria. RAS gene test was performed in 201 patients of entire cohort, and HER2 positivity only presented in patients with RAS wild type (*P* = 0.001) based on both criteria. The result was reasonable due to HER2 and RAS protein are the upstream and down steam of MAP kinase pathway; therefore, HER2 positivity and RAS mutation were mutually exclusive. Moreover, we found HER2 positivity was mainly presented in patients with mismatch repair proficiency (MMR-P), which also can be explained by sporadic MSI. CRC is associated in 60% of tumors with BRAF mutation and BRAF is the downstream of HER2 protein [7]. Previous studies have reported that the HER2 expression was significantly correlated with tumor size, histological differentiation, lymph node metastases, and tumor stage [25–28]. In contrast to these studies, other researchers have found no such association [29, 30]. These studies analyzed the association using the GEA criteria to identify HER2 positivity, and the results were equivocal. However, our results found that based on the HERACLES criteria, HER2 positivity was more common in CRC patients with left-sided, presence of regional lymph node metastasis, advanced stage, and RAS wild type.

To further explore the impact of HER2 on survival based on different scoring systems, we selected patients with stage II-III CRC who receive surgery as initial treatment; the results showed that gender, vascular invasion, perineural invasion, HER2 status, and tumor stage were associated with DFS, yet multivariate analysis showed only tumor stage was the independent prognostic factor according to the HERACLES criteria. No such association between HER2 status and DFS was found based on the GEA criteria, but the *P* value was 0.052. There were only 3 cases in which HER2 status was positive in the GEA criteria and negative in the HERACLES criteria in patients with stage II-III disease. Among them, one patient presented lung recurrence at 13.1 months after surgery, and the rest two did not present recurrence at the time of censor. Therefore, it was reasonable that the results were marginal and HER2 positivity was a factor associated with worse DFS according to the HERACLES criteria. The negative prognostic impact of HER2 overexpression was also be discovered in other studies [19, 27]. The different results between HER2 status and clinicopathological factors, survival in CRC indicated that the HERACLES criteria would be a favorable scoring system for HER2 assessment of CRC.

Considering that anti-HER2 treatment was applied in mCRC, we further evaluated the correlation between HER2 expression and clinicopathological factors in mCRC, and influence on PFS. It is interesting that HER2 status turned out to be the same according to the two criteria. Moreover, all eight cases which present 3+ IHC score were presented in more than 50% of the cells. The results indicated that overexpression of HER2 protein was low in mCRC but it was highly expressed in more than half the tumor cells in our study. Still, HER2 status was found all negative in RAS mutant group. The prognostic role of HER2 in mCRC remains uncertain. Ingold Heppner B et al. [27] considered that although statistically not significant (*P* = 0.208), HER2-positive colorectal carcinomas displayed a tendency to poorer overall survival. We found that HER2 expression had no impact on the PFS of mCRC patients. Although our study did not find a difference in PFS between the HER2-positive and HER2-negative groups, the mean PFS was longer in the HER2-positive group than in the HER2-negative group (23.7 months vs. 15.4 months). It seemed that HER2 positivity played a positive impact on survival of patients with mCRC; however, a negative impact on patients with stage II-III CRC. The exact role of HER2 on the process of CRC molecular pathogenesis need to be further explored.

There were several limitations of this study. First, the data were from a single center, and the results only reflected HER2 expression in southern China due to patients in our hospital mainly came from provinces of south China. Second, we did not perform FISH on cases with an IHC score of 3+ in more than 50% of the tumor cells for both criteria and considered these cases as HER2 positive. Therefore, it was difficult to evaluate the accordance rate of IHC and FISH in cases with IHC score 3+, as well as positive agreement and negative agreement rates. Third, as a result of the limited follow-up time, we did not evaluate overall survival in the entire cohort. Finally, this was a descriptive, retrospective study, and we did not investigate the effects of anti-HER2 treatment in mCRC.

Our study indicated that the frequency of HER2 overexpression or amplification was low in CRC in Chinese population. HER2 status evaluated by the HERACLES criteria showed clinicopathological association and survival impact on CRC, not by the GEA criteria. Additionally, HER2 positivity in mCRC was identical according to both criteria. Our findings provided a rationale for further evaluation of HER2 in CRC based on the HERACLES criteria and the HER2 diagnostic criteria for gastroesophageal adenocarcinoma.

## Electronic supplementary material

ESM 1 (DOCX 33.7 kb)
