# Supplementary material for: Assessment of two different HER2 scoring systems and clinical relevance for colorectal cancer
Source: Virchows Arch. 2019 Nov 13;476(3):391–8. doi: 10.1007/s00428-019-02668-9 (PMC7085476; doi:10.1007/s00428-019-02668-9)
Supplement: Supplementary file 1 — (DOCX 33.7 kb) [file 428_2019_2668_MOESM1_ESM.docx]

**Supplementary Fig. 1** Flow diagram of the patients' selection process and treatments in this study

CRC with HER2 test (n = 1560)

Did not fulfill inclusion criteria

Incomplete data (n = 45)

Histologically-prove CRC with complete data (n = 1515)

Excluded (n = 25)

Lack of tumor tissue for FISH test (n = 5)

Presence of simultaneous carcinoma (n = 11)

Refusal anti-tumor treatment (n = 9)

Eligible patients (n = 1490)

Metastatic CRC (n = 244)

Non-metastatic CRC (n = 1246)

Received surgery (n = 234)

Did not receive surgery (n = 10)

Received surgery (n = 1245)

Did not receive surgery (n = 1)

Neoadjuvant chemotherapy (n = 62)

Neoadjuvant chemoradiotherapy (n = 184)

Patients gained clinical complete remission (n = 1)

No neoadjuvant therapy (n = 999)

Received adjuvant radiotherapy (n = 1)

Received adjuvant chemoradiotherapy (n = 24)

Received adjuvant chemotherapy (n = 589)

Entered follow-up period (n = 385)

next page

Conversion chemotherapy (n = 88) and chemoradiotherapy (n = 22)

Received 5-Fluorouracil-based chemotherapy (n = 99)

Received irinotecan-based chemotherapy (n = 6)

Received irinotecan and 5-Fluorouracil chemotherapy (n = 5)

Along with targeted therapies (Cetuximab: n = 11; Bevacizumab: n = 12)

Did not receive conversion therapy (n = 134)

Received 5-Fluorouracil-based chemotherapy (n = 124)

Received irinotecan-based chemotherapy (n = 6)

Received irinotecan and 5-Fluorouracil chemotherapy (n = 1)

Other chemotherapies (n = 3)

Along with targeted therapies (Cetuximab: n = 2)

Did not present progression disease during follow-up period (n = 117)

Progression disease after first-line treatment during follow-up (n = 127)

Refused further treatment or received best supportive treatment (n = 25)

Received chemotherapy (n = 85)

Immunotherapy (n = 1)

Ablation and other treatments (n = 16)

Along with targeted therapies (Cetuximab: n = 5; Bevacizumab: n = 21; Aflibercept: n = 1)

**Supplementary Table 1** Relationship between the expression of HER2 and the clinicopathological prognostic features in metastatic colorectal cancer

| Variables | All patients  (n = 244) | HER2 negativity  (n = 234) | HER2 positivity  (n = 10) | *P* value |
| --- | --- | --- | --- | --- |
| Gender |  |  |  | 1.0 |
| Male | 150 | 144 | 6 |  |
| Female | 94 | 90 | 4 |  |
| Age (years) |  |  |  | 0.440 |
| ≤ 65 | 193 | 186 | 7 |  |
| > 65 | 51 | 48 | 3 |  |
| Primary tumor location |  |  |  | 0.122 |
| Left-sided | 184 | 174 | 10 |  |
| Right-sided | 56 | 56 | 0 |  |
| Both-sided | 4 | 4 | 0 |  |
| Differentiation grade |  |  |  | 1.0 |
| Well | 4 | 4 | 0 |  |
| Moderate | 226 | 216 | 10 |  |
| Poor | 14 | 14 | 0 |  |
| Metastatic sites |  |  |  | 0.5 |
| Liver only | 112 | 107 | 5 |  |
| Lung only | 18 | 16 | 2 |  |
| Multiple organs | 60 | 57 | 3 |  |
| MSI test |  |  |  | 1.0 |
| No. of cases evaluated | 228 ^a^ |  |  |  |
| MSI-H (dMMR) |  | 10 | 0 |  |
| MSS/MSI-L (pMMR) |  | 212 | 6 |  |
| RAS gene |  |  |  |  |
| No. of cases evaluated | 146 ^b^ |  |  | 0.042 |
| RAS wild-type |  | 81 | 7 |  |
| RAS mutation |  | 58 | 0 |  |
| Ki-67 |  |  |  |  |
| No. of cases evaluated | 211 ^c^ |  |  | 0.489 |
| ≤ 15% |  | 18 | 1 |  |
| > 15% |  | 186 | 6 |  |

^a^16 cases did not receive MSI test; ^b^ 98 cases received RAS gene test; ^c^ 33 cases did not receive Ki-67 test.

*HER2,* Human epidermal growth factor receptor 2; *MSI*, microsatellite instability; *MSI-H*, high-level microsatellite instability; *MMR-D*, mismatch repair deficiency; *MSS*, microsatellite stability; *MSI-L*, low-level microsatellite instability; *MMR-P*, mismatch repair proficiency

**Supplementary Table 2** HER2 status in metastatic colorectal cancer

|  | | The criteria for gastroesophageal adenocarcinoma | | The HERACLES diagnostic criteria | |
| --- | --- | --- | --- | --- | --- |
| HER2 (IHC) | Valid/missing | 244/0 |  | 244/0 |  |
|  | 0 | 167 | 68.4% | 167 | 68.4% |
|  | 1+ | 56 | 23.0% | 56 | 23.0% |
|  | 2+ | 12 | 4.9% | 12 | 4.9% |
|  | 3+ | 9 | 3.7% | 9 | 3.7% |
| HER2 (FISH) | Valid/missing | 12/232 |  | 12/232 |  |
|  | Not amplified | 11 | 91.7% | 11 | 91.7% |
|  | Amplified | 1 | 8.3% | 1 | 8.3% |
| HER2 status | Valid/missing | 244/0 |  | 244/0 |  |
|  | Negative | 234 | 95.9% | 234 | 95.9% |
|  | Positive | 10 | 4.1% | 10 | 4.1% |

*HER2,* Human epidermal growth factor receptor 2; *IHC*, immunohistochemistry; *FISH*, fluorescent *in situ* hybridization

**Supplementary Table 3** Univariate and multivariate analyses of disease-free survival in stage II-III patients treated with surgery according to the HERACLES diagnostic criteria

| Factors | Univariate |  | Multivariate | | |
| --- | --- | --- | --- | --- | --- |
|  | *P* value |  | HR | 95% CI | *P* value |
| Age (≤ 65 vs. > 65) | 0.975 |  |  |  |  |
| Gender (male vs. female) | 0.028 |  | 1.368 | 0.913-2.05 | 0.128 |
| Primary tumor location (left vs. right) | 0.229 |  |  |  |  |
| Differentiation grade (Well-Moderate vs. poor) | 0.06 |  |  |  |  |
| Ki-67 (≤ 15% vs. > 15%) | 0.817 |  |  |  |  |
| HER2 (positive vs. negative) | 0.048 |  | 1.772 | 0.848-3.705 | 0.128 |
| Vascular invasion (present vs. absent) | 0.045 |  | 1.108 | 0.726-1.693 | 0.634 |
| Perineural invasion (present vs. absent) | 0.021 |  | 1.354 | 0.907-2.02 | 0.138 |
| Tumor stage (II vs. III) | 0.001 |  | 1.643 | 1.095-2.466 | 0.017 |

*HER2,* Human epidermal growth factor receptor 2
